# Taming the beast within: resveratrol suppresses colitis and prevents colon cancer

**DOI:** 10.18632/aging.100143

**Published:** 2010-04-30

**Authors:** Lorne J. Hofseth, Udai P. Singh, Narendra P. Singh, Mitzi Nagarkatti, Prakash S. Nagarkatti

**Affiliations:** ^1^ Department of Pharmaceutical and Biomedical Sciences, South Carolina College of Pharmacy, University of South Carolina, Columbia, SC 29208, USA; ^2^ Department of Pathology and Microbiology, School of Medicine, University of South Carolina, Columbia,; SC 29208, USA

**Keywords:** Inflammation, complementary and alternative medicine, SIRT-1, NF-kappaB, inflammatory bowel disease

Resveratrol has generated extensive scientific and public interest in recent years mainly because of its ability to delay aging and prevent age-related diseases. Mild-to-moderate red wine consumption has anti-inflammatory properties, and can reduce the risk of cardiovascular disease and cancer.  The resveratrol content in red wine is often cited to account for this "French paradox".  There is increasing literature suggesting that resveratrol may have anti-aging properties  through the activation of silent mating type information regulation-1 (SIRT-1) [1].  We have now shown that inflammation in the colon down-regulates SIRT-1 and enhances nuclear transcription factor-kappaB (NF-κB) while resveratrol reverses this process [2].  We showed the efficacy of resveratrol in a dextran sodium sulfate (DSS)- mouse as well as in a spontaneous IL-10^-/-^ mouse model of colitis.  Specifically, in this as well as our other study [3], we found that resveratrol attenuated overall clinical scores as well as various pathological markers of colitis.  Resveratrol reversed colitis-associated decreased body weight and colon length; and suppressed colitis-induced inflame-matory markers (iNOS, COX-2, TNF-α) and markers of inflammatory stress (p53 and phosphor-p53-serine 15).  Also, resveratrol suppressed the activation of CD3^+^ T helper cells, and reversed DSS-mediated increases in serum amyloid A, TNF-α, interleukin (IL-6), and IL-1β.  After resveratrol treatment, the percentage of neutrophils, and CD4^+^ T cells in mesenteric lymph nodes (MLN) of colitis mice was restored to normal levels, and there was a decrease in these cells in the colon lamina propria (LP). Likewise, the percentages of macrophages and neutrophils in MLN and the LP of mice with colitis were decreased after resveratrol treatment. Our studies demonstrated for the first time that SIRT-1 is involved in colitis, functioning as an inverse regulator of NF-κB activation and inflammation. Furthermore, our results indicated that resveratrol may protect against colitis through the up-regulation of SIRT-1 in immune cells in the colon.  Inasmuch as, it has also been shown that SIRT1 suppresses intestinal tumor formation [4], and colitis drives colon cancer, we hypothesized that resveratrol suppresses colon cancer associated with colitis.  The azoxymethane (AOM)/DSS mouse model was used to test this hypothesis.  Tumor incidence was reduced from 80% in mice treated with AOM + DSS to 20% in mice treated with AOM + DSS + resveratrol. Tumor multiplicity also decreased with resveratrol treatment. AOM + DSS-treated mice had 2.4 ± 0.7 tumors per animal compared with AOM + DSS + resveratrol, which had 0.2 ± 0.13 tumors per animal.  Together, these data indicated that the beneficial effects of resveratrol during colitis may be mediated through several mechanisms.  However, we believe that the important mechanism may involve the negative regulation of NF-κB activity by SIRT-1; as the NF-κB pathway has been shown to contribute to colitis and colon cancer associated with colitis [5].  Thus, downregulation of SIRT-1 during colitis may induce inflammatory cytokines through the activation of NF-κB which is reversed by resveratrol.  Taken together, our studies have indicated that resveratrol-induced SIRT-1 not only protects against aging but also plays a critical role in the regulation of inflammation that controls colitis and colon cancer.  Thus, resveratrol is a useful, nontoxic complementary and alternative strategy to abate colitis and prevents colon cancer associated with colitis.
